# Society Meetings

**Published:** 1902-04

**Authors:** 


### SOCIETY MEETINGS.

The American Association of Pathologists and Bacteriologists held its second annual meeting at Cleveland, Friday and Saturday, March 28 and 29, 1902, under the presidency of Dr. William T. Councilman, of Baltimore. Papers were read by a number of men distinguished in these branches of medical research, among whom were Drs. Harvey R. Gaylord and Herbert U. Williams, of Buffalo.

---

The International Association of the Medical Press will take place on April 7, 1902, at Monte Carlo, under the protection of his highness, the Prince of Monaco. This meeting should have occurred in September, 1901, but for diverse reasons was postponed several months. At the meeting an effort will be made to organise a permanent bureau for the international medical press—similar to our Associated Press—for the reception and delivery of news items to the various medical journals subscribing to this association.

---

The American Congress of Tuberculosis will hold its third annual meeting May 14, 15 and 16, 1902, at the Hotel Majestic, 72d Street and Central Park, West, in the City of New York, in joint session with the Medico-Legal Society.

---

The American Association of Urologists will hold its first annual meeting on the last day and the day following the annual meeting of the American Medical Association. The officers of the association are: Ramón Guiteras, president; Wm. K. Otis, vice-president; John Van der Poel, treasurer; Ferd. C. Valentine, secretary; A. D. Mabie, assistant secretary.

---

The Buffalo Academy of Medicine held meetings during the month of March, 1902, as follows:

*Stated Meeting.*—Tuesday evening, March 4. Program: The advantage of early surgical intervention in borderland cases, Roswell Park.

*Section on Medicine.*—Tuesday evening, March 11. Program: Observations upon two interesting cases of the local manifestations of hysteria, Prescott Le Breton. The present conception of neurasthenia, H. B. Singer.

*Section on Ophthalmology, Otology, Laryngology and Rhinology.*—Monday evening, March 17. Program: Disease of the skin affecting the nose; illustrated by the stereoscope, Grover W. Wende.

*Section on Pathology.*—Tuesday evening, March 18. Program: The chemic basis of dietetics, A. L. Benedict. A case of tertian malaria, arising in West Seneca, Eugene Horton, Stud. Med. An unusual case of mediastinal tumor associated with obstruction of the thoracic duct and accumulation of chyle in the pleural cavity, H. G. Matzinger. Gastric ulcer and other specimens, H. U. Williams. Primary tumors of the kidney, H. R. Gaylord.

*Section on Obstetrics.*—Tuesday evening, March 25. Program: Ectopic pregnancy, Henry D. Ingraham. Placenta previa, Matthew D. Mann.

---

THE Society of Physicians of the Village of Canandaigua has sent out the following: Annual meeting, January 8, 1903. Address by the retiring president, Dr. A. L. Beahan. Subject, Fracture of the femur. Program: February 6—Origin of cough, O. J. Hallenbeck. March 6—Postpartum hemorrhage, F. E. McClellan. April 3—Treatment of pulmonary tuberculosis O. J. Mason. May 1—Oxyuris vermicularis, D. A. Eiseline. June 5—Mental derangement in the lower animals, D. R. Burrell. September 4—Epidemics, H. C. Buell. October 2—Typhoid fever, M. R. Carson. November 6—Pneumonia in children, J. H. Pratt. December 4—Treatment of cough, J. H. Jewett.

---

THE Western Ophthalmologic and Oto-Laryngologic Association will hold its seventh annual meeting in Chicago, April 10, 11 and 12, 1902. Provisional program: Address by the president, C. R. Holmes, Cincinnati. Relations of the facial nerve to the tympanum, especially in tympanic exenteration (lantern lecture), B. Alex. Randall, Philadelphia. (by invitation). Symposium: Otorrhea. (*a*) The neighboring parts of the middle-ear and their infection, Otto J. Stein, Chicago. (*b*) The diagnosis of meningitis, phlebitis and cerebral abscess following suppuration of the middle-ear, C. Barck, St. Louis. (*c*) What eye symptoms are of value in the localisation of brain diseases, C. Barck, St. Louis. (*d*) When should one operate for the cure of chronic suppurative otitis media? O. Joachim, New Orleans. (*e*) Principles of treatment. Wm. L. Ballenger, Chicago. A new field-of-hearing chart, Derrick T. Vail, Cincinnati. Pneumatic massage in aural practice, Edwin Pynchon, Chicago. Sources of error in functional tests of the ear, A. H. Andrews, Chicago. Thiosinamine and electrolysis in the treatment of tubal obstruction, Jos. C. Beck, Chicago. The lymphatics of the neck, J. Holinger, Chicago. The profession is cordially invited to attend.

